# The Mechanism of Acrylamide-Induced Neurotoxicity: Current Status and Future Perspectives

**DOI:** 10.3389/fnut.2022.859189

**Published:** 2022-03-25

**Authors:** Mengyao Zhao, Boya Zhang, Linlin Deng

**Affiliations:** ^1^State Key Laboratory of Bioreactor Engineering, School of Biotechnology, East China University of Science and Technology, Shanghai, China; ^2^Shanghai Collaborative Innovation Center for Biomanufacturing Technology (SCICBT), Shanghai, China

**Keywords:** acrylamide, neurotoxicity, oxidative stress, apoptosis and autophagy, inflammation, gut-brain axis

## Abstract

Acrylamide (ACR), a potential neurotoxin, is produced by the Maillard reaction between reducing sugars and free amino acids during food processing. Over the past decade, the neurotoxicity of ACR has caused increasing concern, prompting many related studies. This review summarized the relevant literature published in recent years and discussed the exposure to occupational, environmental, and daily ACR contamination in food. Moreover, ACR metabolism and the potential mechanism of ACR-induced neurotoxicity were discussed, with particular focus on the axonal degeneration of the nervous system, nerve cell apoptosis, oxidative stress, inflammatory response, and gut-brain axis homeostasis. Additionally, the limitations of existing knowledge, as well as new perspectives, were examined, specifically regarding the connection between the neurotoxicity caused by ACR and neurodegenerative diseases, NOD-like receptor protein 3 (NLRP3) inflammasome-related neuroinflammation, and microbiota-gut-brain axis signaling. This review might provide systematic information for developing an alternative pathway approach to assess ACR risk.

## Introduction

For decades, acrylamide (ACR) has been widely used in the paper industry, as well as for wastewater treatment and soil conditioning. It is also used in medicine and the textile industry, mainly to synthesize high molecular polymers, such as poly-ACR (1). In 1994, ACR was listed as a class 2A substance by the International Agency for Research on Cancer (IARC) (“most likely carcinogenic to humans”) (2). In 2002, Swedish scientists first discovered the presence of ACR in heat-processed foods rich in asparagine, raising widespread concern (3, 4). In the same year, two articles on the formation mechanism of ACR were published in Nature, confirming that the Maillard reaction was primarily responsible for ACR formation in heat-processed foods, causing concern regarding ACR exposure *via* normal dietary intake (5, 6). In 2015, the European Food Safety Authority (EFSA) issued a survey report on the ACR content in 43,419 food products. The results showed that the average ACR content in fried potato products was as high as 1.0 mg/kg, while the highest ACR content in coffee was 4.5 mg/kg (7). Moreover, the European Commission set the residue limits of ACR in potato crisps at 750 μg/kg and roast coffee and coffee substances at 400–850 μg/kg (8). Therefore, ACR toxicity and its risk to human health require urgent attention due to its abundance in food products and the environment (9).

Acrylamide is responsible for developmental genotoxicity, neurotoxicity, and potentially carcinogenicity, of which neurotoxicity has been confirmed *via* human and animal experiments (10). Therefore, neurotoxicity is closely connected with human health. For decades, results showed similar phenotypical neurotoxicity in various laboratory animals, including dogs, cats, guinea pigs, rabbits, and rodents when repeatedly exposed to ACR levels ranging between 0.5 and 50 mg/kg/day (11–13). These neurological disorders may be caused by covalent adduct formation between highly nucleophilic cysteine and ACR at the active location of the presynaptic neuron. This process deactivates neurons and impacts neurotransmitter transfer, leading to neurotoxicity (14). Moreover, oxidative stress serves as a biochemical and physiological activation signal and is, directly and indirectly, related to the neurotoxicity caused by ACR (15, 16). Although the potential molecular mechanism reported in recent years as underlying ACR-related neurotoxicity is multifaceted, a complete characterization and summary of the comprehensive mechanical system and its impact are still required.

Therefore, this review summarizes the relevant literature published in recent years, discusses the primary potential mechanisms, and provides insight into the axonal degeneration of the nervous system, nerve cell apoptosis, oxidative stress, inflammatory response, and gut-brain axis homeostasis. Furthermore, the existing challenges and research aspects showing potential for examining ACR-induced neurotoxicity are discussed. This review may provide a more complete theoretical foundation to uncover the ACR toxicity mechanism while improving its subsequent adverse impact. This paper offers a fresh perspective on neurotoxicity while furnishing guidance regarding health safety development.

## Exposure to Acrylamide

### Occupational and Environmental Exposure

As a raw material used during industrial production, ACR often enters the water, soil, atmosphere, and other environmental media, damaging human health and severely affecting the nervous system (17). Occupational exposure is one of the main ACR exposure pathways. As early as the 1950s, it was reported that several workers exposed to ACR showed symptoms of poisoning, such as weakness, numbness, leg weakness, and unsteady gait. In recent years, occasional studies have examined ACR neurotoxicity, primarily acute occupational ACR poisoning during construction, coal mining, flocculator manufacturing, and tunnel construction (18). In addition to occupational exposure, ACR exposure can occur *via* drinking water, especially when public drinking water sources are treated using polyacrylamide as a flocculant (19), leading to high ACR concentrations in tap water (20, 21). Moreover, residual ACR is present in cosmetics, packaging materials, and cigarettes, while exposure can also occur *via* direct skin contact and alimentary contact (22–24). Table 1 summarizes the environmental and occupational characteristics of ACR exposure, including routes, estimated doses, typical cases, and threshold values. In summary, extensive research has been conducted involving the potentially harmful effect of ACR in occupational settings and daily life. However, additional studies are necessary to fully understand the chronic toxic effect of ACR and improve risk assessment to protect public health.

**TABLE 1:** A summary of the occupational and environmental exposure to ACR.

	Exposure route	Typical cases	Estimated exposure dose	Threshold value
Occupational exposure	Construction, coal mining, flocculator manufacturing, tunnel construction (18)	In 2001, the hemoglobin (Hb) adduct levels of tunnel workers exposed to ACR were measured, representing the blood biomarker for ACR exposure (120)	17.7 nmol/g globin (163 workers)	∼0.02–0.07 nmol/g globin
		Two workers developed peripheral neuropathy after ACR monomer exposure in a closed environment without sufficient ventilation (121)	/	0.1 mg/mL (EU) (122)
Environmental exposure	Drinking water (19)	In the United Kingdom, ACR was found in tap water in an area where polyacrylamide was used for water treatment (20)	0.75 μg/L	<0.5 μg/L (WHO) (21)
		Plymouth, United Kingdom uses polyacrylamide to treat public drinking water. High ACR concentrations were found in drinking water samples (123)	4.5 μg/L	
	Cosmetics (24)	ACR in cosmetics is absorbed through the skin	1 μg/kg b.w./day	<5 mg/kg (American Cosmetic Raw Material Evaluation Committee) (24)
				Body care products<0.1 ppm Other cosmetic products <0.5 ppm (102)
	Packaging materials (22)	ACR is widely used as a papermaking additive in many paper packages. Tests show that ACR exposure from paper packaging poses a safety risk (124)	0.5–8.8 mg/kg	/
	Cigarettes (7)	Zhang et al. examined 51 local volunteers and further correlated the exposure model with the daily intake of ACR (23)	Non-smokers 1.08 ± 0.51 μg/kg b.w./day Smokers 4.18 ± 1.13 μg/kg b.w./day	/

### Foodborne Exposure

Consumers can be directly exposed to ACR *via* the oral intake of high-carbohydrate foods, such as potato chips, baked cereals, and bread (18). As early as 2010, the JECFA presented data regarding the ACR detected in 12,582 food samples from 31 countries, which included fried potatoes, bread, biscuits, coffee, and other food products. The results indicated the presence of ACR in almost all the analyzed food samples, with the highest levels in fried potato products and coffee (21). In 2011, the JECFA evaluated the dietary intake of ACR in eight representative countries. The findings showed that the average daily intake of the general population was about 1 μg/kg b.w., with the highest consumption at about 4 μg/kg b.w. (25). Moreover, young people tend to consume food products high in carbohydrates more frequently. The average exposure of adolescents aged 10–18 is 0.4–0.9 μg/kg b.w./day, and the high-level exposure is 0.9–2.0 μg/kg b.w./day. For children aged 3–10 the average exposure is 0.9–1.6 μg/kg b.w./day, while the high-level exposure can reach 1.4–3.2 μg/kg b.w./day. Based on body weight, the intake of ACR in children is two to three times higher than that of adults (7). In 2015–2017, a survey report by EFSA showed that the average ACR content in fried potatoes and coffee products remained high at 1 and 5 mg/kg, respectively (7). In 2017, the EU set the benchmark levels for the presence of ACR in food products, with that of French fries (ready-to-eat) at 500 μg/kg and potato dough products at 750 μg/kg. The benchmark level for roasted coffee was 400 μg/kg, while that of instant coffee was 850 μg/kg (26). Therefore, ACR toxicity and its risk to human health require urgent attention due to its abundance in food products and high exposure frequency.

### The Metabolic Pathway of Acrylamide

**FIGURE 1:**
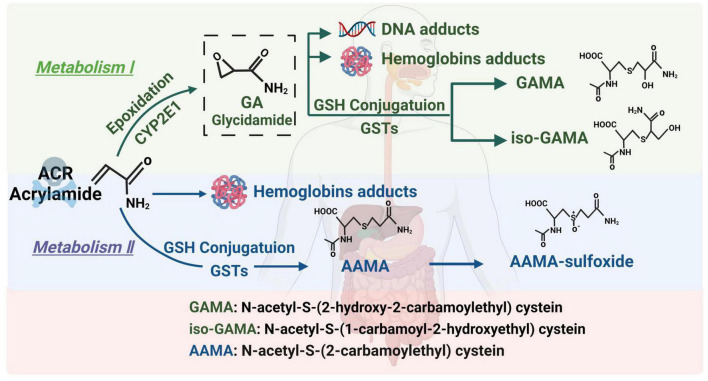
The metabolic pathway of ACR.

Acrylamide is a small-molecule hydrophilic substance. It is absorbed *via* the gastrointestinal tracts of humans and animals and passively diffused to the entire body. ACR can also pass through the blood-brain barrier to directly exert its toxic effect on the nervous system (27). ACR follows two main metabolic pathways in the body (Figure 1). (1) Briefly, an enzymatic reaction occurs when catalyzed by the cytochrome P450 enzyme system, CYP2E1, converting ACR into glycidamide (GA) (28). Studies have found that GA can combine with purine bases on deoxyribonucleic acid (DNA) molecules to form DNA adducts, inhibit the release of neurotransmitters, cause nerve terminal degeneration, damage nerve structures, and display distinct cumulative effects. In addition, the ability of GA to form Hb and DNA adducts is more significant than ACR. Therefore, it is believed that this pathway is the main route of ACR-induced neurotoxicity (29, 30). (2) ACR undergoes biotransformation and is catalyzed by glutathione S-transferase in the liver, combining with glutathione to generate N-acetyl-S-cysteine. It is further degraded into mercapturic ACR acids, which are excreted in the urine. This pathway is mainly responsible for ACR detoxification (31). Glutathione consumption reduces antioxidant levels, leading to excessive active oxygen accumulation and causing oxidative stress and neurotoxicity (32). Various reviews elaborate on the metabolic pathways of ACR. Those published by Koszucka et al. (27), Rifai et al. (28), and Fang et al. (30) are recommended for more details.

### Model and Dosage

Previous studies involving ACR neurotoxicity mostly used a dose of 5–50 mg/kg/day during animal experiments in the in vivo model, with an average dose range of 1–5 mM in various cell lines. Toxic relationship and mechanism investigation mainly established between exposure to high concentrations (>2 mM *in vitro* or 20 mg/kg body weight *in vivo*) of ACR and neurotoxicity.

Barber and LoPachin exposed Sprague Dawley (SD) rats to a dose of 50 mg/kg/day for 28 days to explore ACR neurotoxicity, revealing that the mice displayed significant weight loss and abnormal gait (33). Yu et al. exposed male Wistar rats to doses of 20 and 40 mg/kg/days for 8 weeks, revealing the same phenomenon (34). In 2011, another study exposed adult male SD rats to 50 mg/kg/day ACR for 10 days. The results showed that ACR toxicity was associated with selective nerve terminal damage in the central and peripheral nervous systems (35). Santhanasabapathy and Vasudevan exposed adult Swiss albino male mice to a dose of 20 mg/kg/day for 4 weeks, indicating that ACR promoted microglial and astrocyte activation (36). Recently Guo and Cao exposed adult male SD rats to 40 mg/kg/day ACR for 4 weeks, showing the death of hippocampal neurons and neurotoxicity caused by oxidative stress (37). Moreover, the ACR exposure dose is relatively high in most *in vitro* studies. Human embryonic stem cells (H1hESC) were exposed to 2.5 mM and 5 mM for 24 h, demonstrating that ACR inhibited neuron differentiation based on the oxidative stress response (38). Similarly, Li and Sun exposed rat adrenal pheochromocytoma cells (PC12) to 5 mM ACR for 24 h, revealing oxidative stress-induced cytotoxicity (39). Triningsih and Yang used 5 mM 24 h ACR treatment to explore the relationship between ACR neurotoxicity and autophagy in PC12 cells (40).

Exposure to high ACR concentrations can produce central-peripheral signal transduction obstacles and influence neural development in occupationally exposed humans and laboratory animals (41–43). However, dietary intake is the main source of ACR exposure in humans (4). Several *in vivo* studies established models with a dose below 5 mg/kg/day and exposure times exceeding 60 days to investigate the neurotoxic effect and mechanism of foodborne ACR exposure. For example, SD rats provided with treated drinking water containing ACR dosages of 0, 0.5, or 5 mg/kg/day for 12 months displayed gait abnormality and cognitive dysfunction (44). Zhao et al. orally exposed C57BL/6 male mice to a low ACR dose of 5 mg/kg/day for 60 day (sub-chronic toxicity) to explore its neurotoxicity. This group displayed a slight gait abnormality at the end of the intonation period (45). Similarly, doses (about 1–5 μM) closer to chronic foodborne exposure are relatively rare in *in vitro* exposure models. Zong et al. demonstrated that ACR exposure increased the expression of cytokines and inflammatory markers in BV-2 microglial cells after treatment with a low ACR dose of 5 μM (46). Currently, extensive research has focused on the high-dose neurotoxicity, while chronic foodborne exposure of ACR has not received enough attention. In addition, health risk assessments require information regarding the relationship between exposure, internal brain dosage, and the observed toxic effects. Therefore, further research is needed to clarify and assess the risk of foodborne ACR neurotoxicity.

## Mechanisms of Acrylamide-Induced Neurotoxicity

### Axonal Degeneration

Early research suggests that ACR-induced neurotoxicity may be related to nerve-ending damage in the peripheral and central nervous systems (47). Studies have shown that ACR can change the β-actin, β-tubulin, and other cytoskeletal proteins, destroying the neuron structures to cause neurotoxicity. Subsequent morphological, electrophysiological, and electrochemical research shows that nerve endings represent the primary target of ACR toxicity (48). With the passage of exposure time, the damage is gradually aggravated and eventually leads to axonal degeneration. Chronic ACR poisoning can cause selective peripheral and central nerve fiber degeneration, which initially occurs at the ends of long and large nerve fibers, followed by progressive, continuous proximal axon degeneration (49). Further studies show that ACR affects the levels of actin, motor proteins, and other neuronal proteins, resulting in an insufficient supply of adenosine triphosphate, impairing axonal transport functionality (50).

With the development of mass spectrometry and nuclear magnetic resonance technology, researchers found that ACR, as an electrophilic reagent, can quickly attack the sulfhydryl group on proteins and react with molecular DNA while adduct formation may be a reason for its neurotoxicity (51). In addition, ACR can also attack protein sites containing thiols, interfere with the presynaptic nitric oxide (NO) signaling pathway, damage presynaptic nerve endings, disturb nerve signal transmission, and produce neurotoxicity (52). Moreover, phosphorylated Tau agglomeration causes cytoskeletal instability and neuronal malfunction or even death (53). According to recent research, ACR induces Tau tubulin hyperphosphorylation and brain-derived neurotrophic factor (BDNF) reduction in rat hippocampi, resulting in synaptic damage and spatial cognitive impairment (54). Furthermore, chronic ACR exposure leads to motor dysfunction by significantly degenerating dopaminergic and acetyl cholinergic neurons (55). Kopańska et al. believed that the cholinergic anti-inflammatory pathway was closely related to ACR-induced neurotoxicity. ACR decreased cholinergic conduction and acetylcholine secretion, inhibiting the cholinergic anti-inflammatory pathway and causing a potential systemic inflammatory reaction (56). Moreover, it is worth noting that the cognitive impairment caused by neuron degeneration is a precursor of Alzheimer’s disease (57), while the loss of dopaminergic neurons in the brain is related to Parkinson’s disease, indicating that ACR may be a risk factor for the pathological development of neurodegenerative diseases (58).

### Apoptosis and Autophagy

As a common form of programmed cell death, apoptosis plays a vital role in neurodegenerative diseases (59). Neurocytes, including neurons, astrocytes, and microglia, are essential in maintaining brain function (60). Astrocytes mainly support neurotrophic functionality (61). As the resident immune cells in the central nervous system (62), microglial activation is crucial for collective immune monitoring, but excessive activation leads to the release of pro-inflammatory factors (63). Neurons represent the main executors of nerve impulses and, if damaged, cause cognitive impairment and motor dysfunction (64). Early studies have indicated that ACR can induce the apoptosis of various neurocytes, such as human neuroblastoma SH-SY5Y cells, astrocytoma U1240-MG cells, and rat stellate cells (65, 66). Therefore, ACR may damage brain homeostasis and cause neurotoxicity by inducing nerve cell apoptosis.

Liu et al. conducted a mechanism investigation and found that ACR caused mitochondrial malfunctioning in human astrocytoma cells and BV-2 mouse microglia, activated caspase-9 and its downstream pathway, up-regulated the BCL2-associated X protein (Bax)/B-cell lymphoma-2 (Bcl-2) ratio, and induced mitochondrial-dependent apoptosis and neurotoxicity (67). Moreover, other signaling pathways are also involved in ACR-induced apoptosis. Mitogen-activated protein kinase (MAPK), a serine-threonine protein kinase, can control many cell activities, such as apoptosis and the proliferation and expression of signal-regulated protein kinase [extracellular regulated protein kinases (ERK), c-Jun N-terminal kinase (JNK), and p38 protein] genes (68, 69). The inactivation of ERK and activation of JNK and p38 are essential for inducing apoptosis. Tabeshpour et al. found that intraperitoneal ACR injection reduced the p-ERK/ERK ratio while increasing the Bax/Bcl-2, p-JNK/JNK, and p-p38/p38 ratios in the cerebral cortexes of rats, indicating that the MAPK signaling pathway promoted ACR-induced apoptosis (70). Additionally, nuclear factor-κB (NF-κB) regulates a variety of target genes, such as proliferation and apoptosis, while the NF-κB signal is prone to crosstalk and affects several signaling pathways (71). ACR can promote apoptosis *via* the MAPK-driven NF-κB signaling pathway, ultimately resulting in cytotoxicity (72). The nuclear factor E2-related factor-2 (Nrf2) family represents a transcriptional factor that regulates the redox state of cells and participates in the coordination of adaptive responses to various stimuli (73). Pan et al. found that ACR activated the Nrf2 and MAPK pathways in PC12 cells. The MAPK served as an upstream regulator to control the nuclear translocation of Nrf2, exhibiting an antioxidative effect (74). It is worth noting that MAPK could control both the Nrf2 and NF-κB cascades as an upstream factor and facilitate dual-direction regulation (75). Consequently, crosstalk between the NF-κB, MAPK, and Nrf2 signaling pathways is vital during ACR-induced apoptosis and neurotoxicity.

**FIGURE 2:**
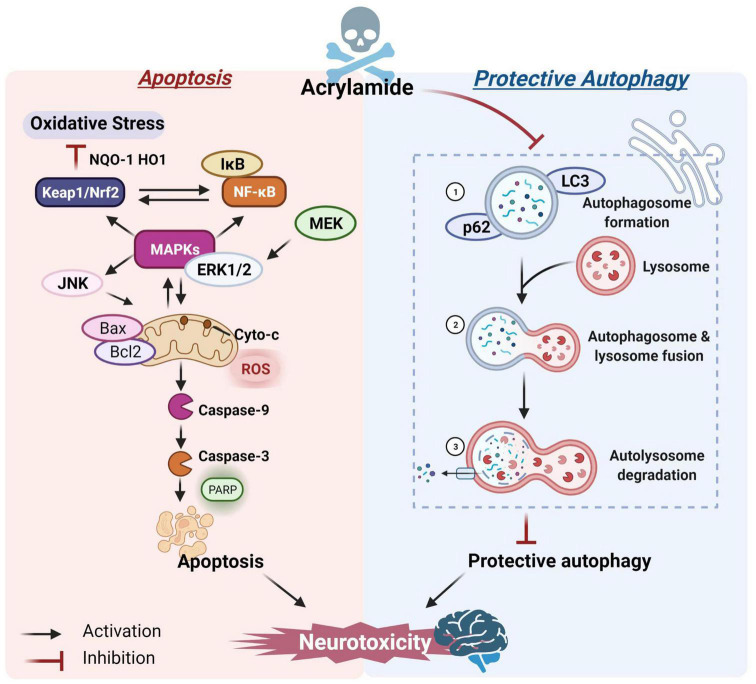
The potential mechanism of ACR-induced apoptosis and autophagy.

Lysosome-mediated protective autophagy is essential for maintaining intracellular redox balance and is crucial for clearing damaged cell proteins and organelles, as well as regulating cell death and survival (76). However, high ACR doses may inhibit autophagy. For example, Liu et al. revealed that sub-chronic exposure to ACR could block autophagy flow by inhibiting lysosomal protease D in the hippocampus, decreasing the inflammasome clearance, and inducing local inflammation (44), which might be a decisive factor in ACR neurotoxicity. Song et al. reported that ACR caused autophagic marker microtubule-associated protein 1 light chain 3-II (LC3-II) and p62 accumulation, suggesting that ACR might inhibit cellular autophagy (77). However, autophagy flow is a finely regulated process and includes (1) autophagosome formation, (2) autophagosome and lysosome fusion, and (3) autolysosome degradation (78). Moreover, the crosstalk affiliation between apoptosis and autophagy is critical for brain health maintenance and central nervous system pathogenesis (79, 80). A study by Deng et al. confirmed the immunofluorescence and co-localization positions and co-expressed intensity of key autophagic markers during the three-autophagic process. The ACR-induced autophagosome accumulation could probably be ascribed to blocked autophagic flux, preventing the autophagosomes from combining with lysosomes. Additional information substantiating the connection between cellular apoptosis and autophagic flux showed that restricting the protective autophagy caused by ACR further promoted the initiation of apoptosis (Figure 2)(81).

### Oxidative Stress

The brain is more vulnerable to oxidative stress than other organs (82, 83), and most neurocyte components can be oxidized and damaged (84). Recent studies have shown that oxidative stress may be involved in the occurrence and development of neurodegenerative and chronic diseases (such as stroke, diabetes, Parkinson’s, Alzheimer’s, and other diseases) (85). Although reactive oxygen species (ROS) are produced during the normal physiological process, excessive production inhibits antioxidant reductase activity and disrupts redox balance, leading to oxidative stress damage (36). ACR-induced neurotoxicity typically manifests as intracellular glutathione depletion (86), indicating that oxidative stress may play a significant role in ACR neurotoxicity.

Mitochondria are essential organelles for intracellular energy metabolism and redox system regulation. Zhao et al. indicated that ACR disrupted the activity of the mitochondrial electron transport chain complexes, I, III, IV, and V, resulting in mitochondrial membrane swelling, the collapse of inner membrane potential, obvious electron transfer disorder, and electron leakage, leading to ROS accumulation (87). Liu et al. also found that ACR significantly increased the ROS level in the BV-2 microglial cell line, leading to oxidative stress, mitochondrial damage, and functionality loss while increasing the expression of caspase-dependent apoptosis-related genes (67). Damage to the mitochondrial structure and functional loss are hallmarks of endogenous apoptosis, activating the Bcl-2 protein family (88, 89). These results suggest that ACR-mediated mitochondrial dysfunction and oxidative stress injury are crucial factors leading to caspase-cascade activation and cellular apoptosis. Moreover, subsequent studies have revealed ACR-induced crosstalk in multiple signaling pathways during oxidative stress response and mitochondrial dysfunction. The Nrf2/NF-κB pathway in astrocytes and microglia was sequentially activated, gradually causing glutathione consumption, ROS accumulation, mitochondrial damage, and neuroinflammation (16).

Oxidative stress is deemed a significant predisposing factor in the pathogenesis of ACR-induced neurotoxicity, of which an increase in ROS accumulation or induced oxidative stress in the brain is only phenotypical. Of fundamental importance is linking these redox shifts to various signaling pathways and explaining how the changes occur. For example, research should explore (1) how oxidative stress arises *via* ACR in the brain, (2) how it is neutralized, (3) which typical signaling or genetic factors may trigger ACR-induced neurotoxicity development, and (4) how this impacts inflammation or autoimmunity in the central nervous system. Consequently, novel, more precise, and effective therapies can then be established to better understand how oxidative stress drives the underlying neurotoxic mechanisms.

### Inflammation

The immune system recognizes external stimuli like pathogen-associated molecular patterns (PAMPs) and damage-associated molecular patterns (DAMPs) *via* pattern recognition receptors (PRRs). Furthermore, cellular PRRs, such as the nucleotide oligomerization domain (NOD)-like receptor (NLR), can activate the downstream immune-inflammatory response. Of these, NOD-like receptor protein 3 (NLRP3) in the NOD subfamily has been widely studied due to its unique response mechanism to a variety of stimuli (90). Research has shown that misfolded proteins like α-synuclein and β-amyloid can activate NLRP3 inflammasomes in the microglia, which may participate in neurodegenerative disease development, such as Parkinson’s and Alzheimer’s diseases (91). Mild cognitive impairment and the early symptoms of Alzheimer’s disease are similar to that of chronic neurotoxicity induced by ACR, suggesting the involvement of NLRP3 inflammasomes.

The primary manifestation of immune inflammation involves inflammatory factor secretion, such as tumor necrosis factor-α (TNF-α), interleukin-6 (IL-6), interleukin-18 (IL-18), and interleukin-1β (IL-1β) (92). Previous research by Zhao et al. found increased TNF-α, IL-6, and IL-1β levels in the primary microglia during the later stage of ACR exposure, further confirming the involvement of the immune-inflammatory response in ACR-induced neurotoxicity (16). Recent studies have shown that the NLRP3 inflammasome level was up-regulated in the hippocampi of rats after oral ACR administration, further increasing the expression levels of pro-inflammatory cytokines, IL-1β, IL-6, and IL-18 (46). Liu reported that inflammatory factors were released *via* the microglial activation facilitated by persistent ACR exposure. NLRP3 inflammasome activation increased the level of IL-1β, raising the levels of other inflammatory factors directly responsible for neuronal injury in the cerebrums of rats (44). Moreover, treatment with the NLRP3 inhibitor (MCC950) or NLRP3 siRNA safeguarded BV-2 microglial cells against the cytotoxicity produced by ACR while reversing NLRP3 inflammasome activation and the subsequent inflammatory response (93). Similarly, in mice exposed to MCC950, NLRP3 knockout intervention provided protection against the neurotoxic damage caused by ACR by restricting Nrf2 antioxidant pathway activation and neuroinflammation (93). Therefore, NLRP3 inflammasomes participate in the neurotoxicity facilitated by ACR and show promise as a target to improve therapeutic strategies.

### Microbiota-Gut-Brain Axis Homeostasis

The brain-gut axis, which is linked by the immune system, is closely associated with mental disease and neurodevelopmental disorders (94). The mechanism underlying the influence of the intestinal barrier and intestinal flora on brain function may include synapse formation regulation (95), neuronal signaling activation, ROS formation inhibition (96), and blood-brain barrier function regulation *via* secondary metabolites (97).

Recent studies suggest that brain-gut axis homeostasis may be another important cause of ACR-induced neurotoxicity. ACR can facilitate circadian cognitive damage and spatial memory impairment by downregulating the protein expression of the circadian rhythm protein (Clock) and BDNF in mice (98). Moreover, ACR increases intestinal permeability, decreases tight junction protein (Occludin) expression, and increases the lipopolysaccharide (LPS) content in the intestine and serum, while the expression of pro-inflammatory cytokines, IL-6, and IL-1β are significantly up-regulated (98). Excessive LPS and inflammatory factors in the blood can induce an immune-inflammatory response, destroy the close blood-brain barrier connection, and aggravate neurogenic inflammation, damaging the neurons and leading to memory impairment (98). Therefore, ACR may interfere with the communication between the intestine and brain *via* the brain-gut axis, leading to circadian rhythm dysfunction, further promoting neurotoxicity development. In addition, ACR can change the diversity of flora in rat feces, reduce the abundance of some beneficial bacteria, and significantly increase the abundance of pathogenic bacteria, resulting in an intestinal flora imbalance and reduced short-chain fatty acids (SCFA) production, further stimulating neurotoxicity (99).

The brain-gut axis attracts increasing attention for the physiological and biological exploration of neurodegenerative, age-related, and neurodevelopmental diseases. Considering the close relationship between ACR toxicity and induced neurodegenerative disorders, the underlying neurotoxic mechanism *via* brain-gut axis homeostasis requires further investigation.

## Conclusion: The Role of Multiple Signaling Pathways in Acrylamide-Induced Neurotoxicity

**FIGURE 3:**
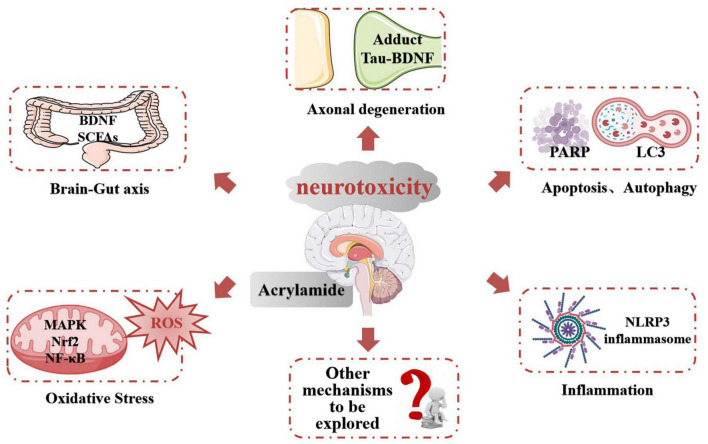
The potential mechanism of ACR-induced neurotoxicity.

Acrylamide neurotoxicity has attracted extensive research attention globally. These reports confirmed that exposure to ACR can lead to neurological disorders like gait abnormality, cognitive impairment, and learning deficiencies. The representative effects and signaling pathways of the neurotoxicity caused by ACR are listed in Table 2. Developing research has gradually proposed a hypothesis regarding the neurotoxic mechanism of ACR and can be summarized as follows (Figure 3): (1) Axonal degeneration, neuronal deficits, and DNA-protein adduct formation are primarily responsible for ACR-induced neurological disorders. (2) Oxidative stress is a typical response caused by ACR and may be associated with mitochondrial malfunction resulting from ACR in conjunction with calcium dyshomeostasis. (3) Multiple neural pathways involving apoptosis, autophagy, and inflammation are attributed to ACR-induced neurotoxicity. (4) Brain-intestinal axis homeostasis and circadian rhythms also participate in ACR-induced neurotoxicity.

**TABLE 2:** A summary of the representative neurotoxic effect of ACR in different models.

No.	Model	ACR dose	Time	Endpoint	Potential mechanism	References
1	PC12 cells	0.6 mM 1.25 mM 2.5 mM 5 mM	24 h	Oxidative stress	ROS↑ MDA↑ GSH↓; HO-1, NQO-1↑. NF-κB (IκBα, p65), ERK1/2, JNK, p38↑. The MAPK pathway is an upstream NF-κB and Nrf2 pathway regulator.	(32)
				Inflammation	TNF-α, IL-6, COX-2↑	
2	Zebrafish (*Danio rerio*)	0.75 mM	3 days	Oxidative stress	ROS↑ MDA↑ GSH↓. Zebrafish exhibit gait abnormalities and negative scototaxis.	(125)
3	H1 hESC cells	2.5 mM 5 mM	24 h	Apoptosis Oxidative stress	SOX2, TUJ1, GFAP, CTIP2, SOX9↓. MAPK, Nrf2↑. FTL, GCLC, GCLM, SLC7A11, HMOX1↑. Caspase-6, caspase-9, c-FOS↑. Stimulated Tau hyperphosphorylation and suppressed neuronal differentiation.	(38)
4	PC12 cells	0.6 mM 1.25 mM 2.5 mM 5 mM	24 h	Apoptosis	Pro-caspase-3, pro-caspase-9, Bcl-2↓. JNK, p-38, Bax, Bax/Bcl-2, cleavage-caspase-9, cleavage-caspase-3↑. Vacuole formation and structural rarefaction cause abnormal mitochondria.	(74)
				Oxidative stress	ROS↑ MDA↑ GSH↓. Nrf2, MAPKs↑. The generation of GSH is reduced *via* Nrf2 knockdown, while the MDA levels are increased.	
5	BV-2 cells	0.5 mM 1 mM 2 mM	24 h	Apoptosis	BDNF, Bcl-2/Bax, p-Akt / Akt↓. Cyto-c, cleavage-caspase-9, cleavage-caspase-3, PARP↑. Decreases mitochondrial respiration and anaerobic glycolysis.	(67)
				Inflammation	iNOS, NO↑.	
6	Male SD rats	0.5 mg/kg/day 5 mg/kg/day	12 months	Inflammation	IL-6, TNF-α, IL-10↑. SYN1, SYP, PSD95↓. NLRP3, caspase-1, IL-1ß↑. Chronic ACR exposure induces neuron lesions and synaptic impairment and activates NLRP3 inflammasomes.	(44)
				Oxidative stress	MDA↑.	
7	PC12 cells	5 mM	24 h	Autophagy	Becline-1, LC3-II, p62↑. ACR induces neurotoxicity *via* activation of PKCs (α, δ) and ERK and inhibition of AMPK.	(40)
8	Male C57BL/6 mice	20 mg/kg/day	4 weeks	Autophagy	ATG4B, LC3-II, Cathepsin D, LAMP2a ↑. Trx-1 siRNA enhances ACR-induced autophagy by regulating ITGAV.	(126)
	PC12 cells	1 mM, 2 mM 4 mM, 8 mM	24 h			
9	Male C57/BL6J mice	Drinking water containing 0.003% ACR	16 weeks	Brain-gut axis inflammation	Bmal1, Clock, SNAP-25, PSD-95, ZO-1, Occludin↓. IL-10, COX-2, TNF-α, COX-2↑. ACR potentially suppresses circadian, impacting cognitive function in the brain.	(98)
10	Male SD rats	50 mg/kg/day	3–28 days	Neurotransmitter dysfunction	Neurological toxicity and weight loss. ACR-cysteine adduct (CEC), 7S SNARE core complex↑. ACR can facilitate adduct formation in the presence of SNARE and NSF proteins (both containing cysteine residues).	(33)
11	Male Wistar rats	20 mg/kg/day 40 mg/kg/day	8 weeks	Axon degeneration	Abnormal gait and nerve damage. The calibers of myelinated axons↓. NF-L, NF-M↑. Accumulation of neurofilaments (NFs).	(34)

## Future Perspectives

Studies are increasingly concentrating on the neurological impact and various underlying mechanisms of ACR. However, despite extensive research involving multiple models, fundamental questions persist, requiring resolution. This section discusses future research from three perspectives, requiring further attention. These insights may further prompt the development of alternative approaches examining ACR risk factors.

### Acrylamide and Neurodegenerative Diseases

As early as 2008, Lopachin et al. suggested that the onset and progression of some neuropathogenic processes, like Parkinson’s and Alzheimer’s diseases and amyotrophic lateral sclerosis, are accelerated by environmental exposure to some type-2 alkenes, such as ACR (100). Moreover, a recent epidemiological study involving 2,534 elderly non-smoking Chinese men indicated that ACR exposure was related to a mild cognitive decrease and a higher risk of poor cognition in four years (101). Extensive research has shown that the various neurotoxic syndromes due to ACR exposure and neurodegenerative diseases, including biochemical changes and neuropathic events in the brain and spinal cord, are similar (102, 103). The inflammatory response and oxidative stress, as well as the related activated signaling pathways present in both neurodegenerative diseases and ACR-induced neurotoxicity, were investigated to clarify the underlying mechanism (13, 104, 105). Therefore, a close connection may exist between the neurotoxicity caused by ACR and neurodegenerative diseases.

However, despite existing comparative studies, some fundamental questions remain. (1) Discoveries regarding ACR-related neurotoxic mechanisms require further investigation based on the neuropathological process of neurodegenerative diseases. (2) How ACR promotes the neuropathological process of neurodegenerative diseases and whether ACR can induce the expression of neurodegenerative pathogenic factors like β-amyloid protein aggregation in the brain remain unclear. However, this presents a fascinating research topic for the future. (3) Clarification is required regarding the existence of a shared target or signaling connection during ACR-induced neurotoxicity and pathological neurodegenerative processing, such as innate immune receptors, toll-like receptor-4, and NLRP3, as well as their signaling interaction. Therefore, more evidence is required. (4) These challenges necessitate a more in-depth understanding of the mechanisms underlying the neurotoxicity caused by ACR. The knowledge regarding the mechanism can be used to develop novel strategies or inhibitors to reduce and block the targets required to manifest adverse ACR effects.

### Nucleotide Oligomerization Domain-Like Receptor Protein 3 Inflammasome-Related Immune Inflammation

**FIGURE 4:**
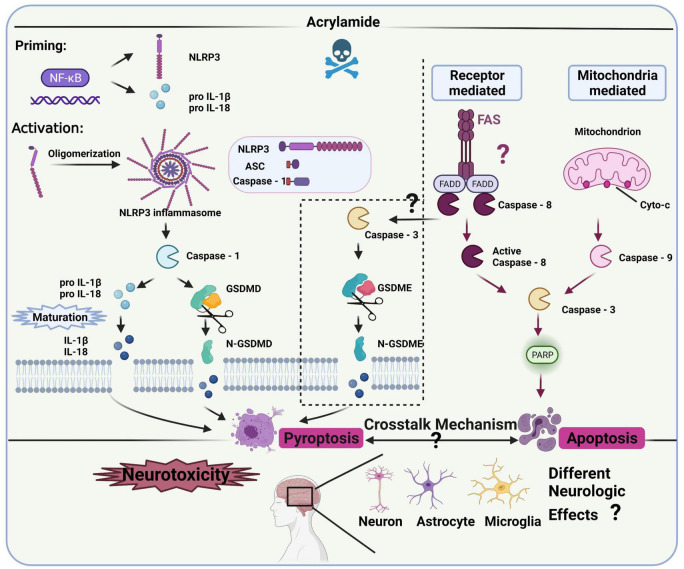
NLRP3 inflammasomes and the related pathway in ACR-induced neurotoxicity.

Several studies have clarified the role of inflammation in the neurotoxicity caused by ACR (36, 72), revealing a connection with NLRP3 inflammasomes (93). However, the specific regulatory role and mechanism of NLRP3 inflammasomes in ACR-induced neurotoxicity require further clarification (Figure 4).

Canonical NLRP3 inflammasomes contain effector protein caspase-1, adaptor protein ASC (a speck-like protein containing a CARD, associated with adaptor molecule apoptosis), and sensory protein NLRP3 (106), while it requires priming and activation to become operational. The priming process includes pro-IL-18, pro-IL-1β, and NLRP3 expression, the initiation of which occurs *via* the NF-κB signaling pathway (107, 108). The activation process allows inflammasome complex assembly, facilitating pro-caspase-1 cleavage to obtain active caspase-1. Then, mature IL-18 and IL-1β are released *via* pro-IL-18 and pro-IL-1β cleavage (44). Additionally, the N-terminal domain of gasdermin D (GSDMD) is released *via* activated caspase-1 cleavage, forming pores in the plasma membrane to rapidly release mature IL-18 and IL-1β, which may cause pyroptosis (109). Besides the canonical pathway, caspase-3 and gasdermin E (GSDME) can also facilitate the release of mature IL-18 and IL-1β while inducing inflammatory cell death, known as alternative pyroptosis in many neurodegenerative diseases and inflammatory response models (110, 111). However, whether the activated caspase-3-IL-1β/IL-18-GSDME signaling contributes to ACR-induced pyroptosis and neurotoxicity remains unknown. Therefore, the pyroptosis-related pathway representing the main signal cascade (NLRP3-ASC-caspase-1-IL-1β/IL-18-GSDMD or caspase-3-IL-1β/IL-18-GSDME) induced by ACR and its neurological impact requires exploration. Furthermore, future studies should also investigate which cascade is activated first during ACR exposure and whether the activation is time-dependent. Additionally, both pyroptosis and apoptosis are involved in programmed cell death but are differentiated *via* morphology (112). ACR can induce pyroptosis and apoptosis with phenotypical traits (109), while caspase-3 represents the effector protein in both the mitochondrially mediated apoptosis pathway and caspase-3-IL-1β/IL-18-GSDME cascade. How ACR dosage, period, and various other factors affect the functionality of caspase-3, as well as the synergistic effect of pyroptosis and apoptosis on the neurological mechanism, require further exploration.

Given the role of inflammasome signaling in the ACR-induced neurotoxic conditions mentioned above, researchers should explore the crucial biomarkers in the pathways to develop specific inhibitors against ACR-induced neurotoxicity. In this case, the correlation between oxidative stress and neuroinflammation should be further refined. For example, how the specific modification of cellular oxidative stress impacts neuroinflammation warrants further attention. Clarification is necessary regarding the connection between mitochondrial dysfunction and inflammasome signaling and whether ion efflux and energy metabolism contribute to this association. Moreover, the potential biomarkers in the NLRP3-related pathway like NLRP3, ASC, caspase-1, GSDMD, and the alternative pyroptosis pathway like caspase-3 and GSDME, should be further confirmed *in vitro* and *in vivo*. An adequate transgenic model must be employed to further verify the NLRP3-related biomarkers and investigate their mechanistic role.

Microglial cells (such as the BV-2 cell line) are generally considered central neuron system immune cells and are activated during the inflammatory response. However, future studies should compare the functional response diversity of neurons, astrocytes, and microglia using different *in vitro* neurocyte models to verify the pathways involved in neuroinflammation while assessing their contribution to ACR neurotoxicity. Another challenge involves achieving cell-cell communication between neurons and glia, specifically concerning the microglia-astrocyte-neuron impact on the neuroinflammation produced by ACR. This interaction may be explained using a co-cultural model.

### Microbiota-Gut-Brain Axis Signaling

The gut-brain axis is deemed an essential pathway for physiological regulation and communication, while gut microbiota are seemingly crucial in this interactive relationship (113). Several pathways are involved in microbiota-gut-brain axis signaling and include the immune system, neurochemical signaling host recruitment, the vagus nerve and direct enteric nervous system routes, and bacterial metabolite production (114–117).

**FIGURE 5:**
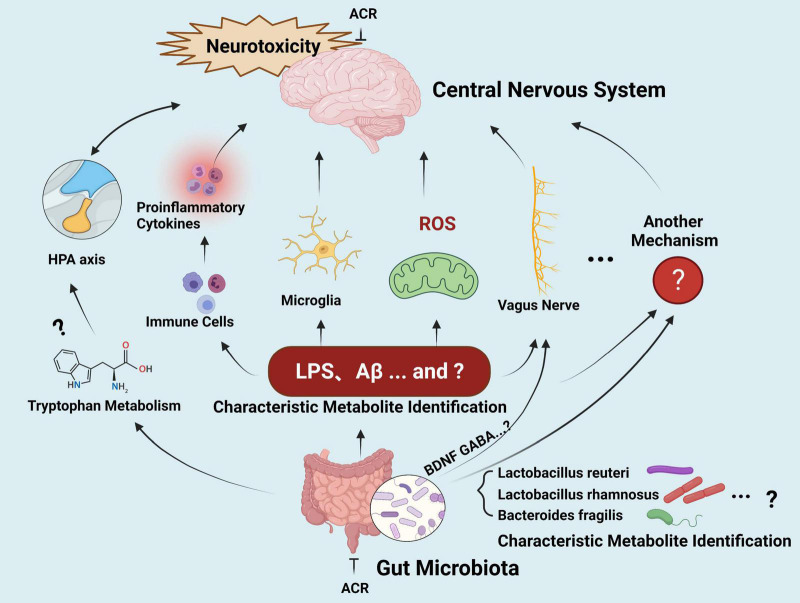
The gut-brain axis in ACR-induced neurotoxicity.

Altered gut microbial profiles and signaling have been described in various neurological disorders, such as Parkinson’s and Alzheimer’s diseases (118, 119). Although the similar neurological impact has prompted some studies to investigate the connection between ACR-induced neurotoxicity and microbiota-gut-brain axis signaling in recent years, systematic studies involving this topic are still lacking. In general, the hypothetical effect of microbiota-gut-brain axis signaling induced by ACR occurs *via* the gut bacterial metabolites of a defective gut barrier, leading to a systematic inflammatory response. This process impairs the blood-brain barrier, ultimately leading to neural damage and deterioration (Figure 5).

However, these hypotheses should be investigated further. (1) Screening and identifying the microbiome of a specific type of putative bacterium (biomarkers) related to ACR-induced neurotoxicity deserves further attention. (2) Further exploration is required regarding potential neurotoxic molecule production, such as amyloid proteins, LPS, and additional secondary metabolites that can reach the central neuron system *via* systemic circulation to activate microglia and ROS signaling. Furthermore, research should clarify whether ACR exposure can modulate the metabolism of normal functional neurotransmitters, such as serotonin and tryptophan, and whether the behavior impacted by ACR-induced gut microbiota and those relying on intact serotonergic neurotransmission overlaps. The underlying crosstalk mechanisms require further elucidation and may be associated with the gut microbial capacity to regulate the metabolism of the host tryptophan in conjunction with the kynurenine pathway. (3) The communication between the brain and the gut during ACR exposure requires clarification. The release of cytokines *via* the immune response represents the most common assumption during the gut-brain communication mechanism. Other influencing routes include the vagus nerve, hypothalamic-pituitary-adrenal axis, and secondary microbial metabolites, while neuromodulators and neurotransmitters may also be involved. However, the details regarding the signaling pathways and crucial biomarkers for communication require further investigation. (4) Limited epidemiological-related data is available, and it is unclear whether any common changes in the gut microbiota are evident in high-risk individuals during ACR exposure. Moreover, whether common microbiota biomarkers are evident in high-risk exposed individuals and neurodegenerative disease patients requires elucidation. During the extended journey from the gut microbiota to the brain, many connections are still unidentified. If this challenge can be resolved, the microbiota-gut-brain axis can facilitate new therapeutic strategies for ACR-induced neurotoxicity and various neurological conditions.

## Author Contributions

MZ: conceptualization, original draft preparation, writing – review and editing, project administration, and funding acquisition. BZ: original draft preparation, especially in summarizing the table and creating the flow chart of the signaling pathways, and writing – review and editing. LD: writing – review and editing and validation. All authors read and approved the final manuscript.

## Conflict of Interest

The authors declare that the research was conducted in the absence of any commercial or financial relationships that could be construed as a potential conflict of interest.

## Publisher’s Note

All claims expressed in this article are solely those of the authors and do not necessarily represent those of their affiliated organizations, or those of the publisher, the editors and the reviewers. Any product that may be evaluated in this article, or claim that may be made by its manufacturer, is not guaranteed or endorsed by the publisher.

## Abbreviations

ACR: acrylamide
ASC: apoptosis-associated speck-like protein containing CARD
Bax: BCL2-associated X protein
Bcl-2: B-cell lymphoma-2
BDNF: brain-derived neurotrophic factor
Caspase-1: cysteinyl aspartate specific proteinase 1
DAMPs: damage-associated molecular patterns
DNA: deoxyribonucleic acid
EFSA: European Food Safety Authority
ERK: extracellular regulated protein kinases
EU: European Union
GA: glycidamide
GSDMD: gasdermin D
GSDME: gasdermin E
GSH: glutathione
Hb: hemoglobin
IARC: International Agency for Research on Cancer
IL-1 β: interleukin-1 β
IL-6: interleukin-6
IL-18: interleukin-18
JECFA: Joint FAO/WHO Expert Committee on Food Additives
JNK: c-JunN-terminal kinase
LC3-II: microtubule associated protein 1 light chain 3-II
LPS: lipopolysaccharide
MAPK: mitogen-activated protein kinase
MDA: malondialdehyde
Nrf2: nuclear factor E2-related factor-2
NF- κ B: nuclear factor-kappa B
NO: nitric oxide
NLR: nod like receptor
NLRP3: NOD-like receptor protein 3
8-OHdG: 8-hydroxy-2-deoxyguanosine
PAMPs: pathogen-associated molecular patterns
PRRs: pattern recognition receptors
ROS: reactive oxygen species
SCFA: short-chain fatty acids
SD: Sprague Dawley
TNF- α: tumor necrosis factor- α
WHO: World Health Organization.
